# Vitamin D Status Is Associated with Modifiable Lifestyle Factors in Pre-Adolescent Children Living in Urban Kuala Lumpur, Malaysia

**DOI:** 10.3390/nu13072175

**Published:** 2021-06-24

**Authors:** Winnie Siew Swee Chee, Chung Yuan Chang, Kanimolli Arasu, Soon Yee Wong, Shu Hwa Ong, Wai Yew Yang, Megan Hueh Zan Chong, Meenal Mavinkurve, Erwin Jiayuan Khoo, Karuthan Chinna, Connie M. Weaver

**Affiliations:** 1 Department of Nutrition & Dietetics, School of Health Sciences, International Medical University, No. 126, Jalan Jalil Perkasa 19, Bukit Jalil, Kuala Lumpur 57000, Malaysia; changchungyuan@student.imu.edu.my (C.Y.C.); KanimolliArasu@imu.edu.my (K.A.); soonyeewong@student.imu.edu.my (S.Y.W.); OngShuHwa@imu.edu.my (S.H.O.); waiyew_yang@imu.edu.my (W.Y.Y.); megan_chong@imu.edu.my (M.H.Z.C.); 2 Department of Paediatrics, School of Medicine, International Medical University, Jalan Rasah, Seremban 70300, Malaysia; meenalmavinkurve@imu.edu.my (M.M.); jiayuan_khoo@imu.edu.my (E.J.K.); 3 Faculty of Health & Medical Sciences, School of Medicine, Taylor’s University, No. 1, Jalan Taylor’s, Subang Jaya 47500, Malaysia; karuthan.chinna@taylors.edu.my; 4 Distinguished Professor Emerita, Purdue University, West Lafayette, IN 47907, USA; weaverconnie1995@gmail.com

**Keywords:** serum 25(OH)D, pre-adolescent children, vitamin D status, lifestyle factors

## Abstract

Studies on vitamin D status and its determinants in growing children in countries with ample sunshine such as Malaysia have been limited. The aim of our study was to determine factors associated with serum 25(OH)D concentrations such as lifestyle, dietary intake, anthropometry, and body composition in 243 pre-adolescent Malaysian children from low-income families living in Kuala Lumpur. This cross-sectional study measured bone density and body composition using dual-energy X-ray absorptiometry (DXA), while serum 25(OH)D was measured using LC–MS/MS. Time spent outdoors, body surface area exposed to sunlight, dietary intake, and physical activity level were assessed using questionnaires. Multiple linear regression and stepwise analysis were performed to identify significant predictors for serum 25(OH)D. About 69.4% had 25(OH)D < 50 nmol/L, and 18.9% were vitamin-D-deficient with 25(OH)D < 30 nmol/L. Girls had a nine-fold higher prevalence of vitamin D deficiency than boys. Body surface area exposed to sunlight, Sun Index, and fat mass were significant predictors of 25(OH)D concentrations in this population. Modifiable lifestyle factors such as sun exposure and reducing obesity are important public health guidance to ensure optimal vitamin D status in these children.

## 1. Introduction

A high prevalence of vitamin D inadequacy is being reported in infants, children, adolescents, and adults worldwide [1,2]. Epidemiologic data on vitamin D inadequacy in children are limited in most countries, but available data have shown that vitamin D deficiency ranges from 10% to 80% in Asian infants, children, and adolescents who live in tropical and sub-tropical countries [3].

Vitamin D status is defined by serum 25(OH)D, and the cut-off to define vitamin D deficiency has been a matter of debate in the past decade. For example, the US Institute of Medicine (now known as the National Academy of Medicine (NAM)) set the dietary reference intake for vitamin D intake based primarily on the integration of bone health outcomes with evidence concerning 25(OH)D levels. The IOM/NAM suggested that a level of 16 ng/mL (40 nmol/L) meets the needs of approximately half the population (median population requirement or estimated average requirement (EAR), and a level of at least 20 ng/mL (50 nmol/L) meets the needs of at least 97.5% of the population (akin to the recommended dietary allowance (RDA) [4]. The Global Consensus Group recommended similar cut-off values of >50 nmol/L (20 ng/mL) as sufficient and <30 nmol/L (12 ng/mL) as deficient for the prevention of nutritional rickets [5]. Previous reports in Malaysia have used cut-off values of >50 nmol/L to define vitamin D status [6,7].

Vitamin D potentially affects a wide range of acute and chronic conditions including infectious disease, asthma, and allergies during childhood [8], but a low vitamin D status is a concern for this age group, primarily because vitamin D plays a critical role in calcium homeostasis and skeletal development. The active form of vitamin D, i.e., 1,25-dihydroxyvitamin D or calcitriol, binds to either the vitamin D-receptor or a responsive gene, i.e., calcium binding protein, and facilitates calcium absorption [9,10]. A low 25(OH)D status leads to reduced intestinal calcium absorption, increased serum PTH concentration, and increased bone loss [10]. Children have higher calcium needs and require a positive calcium balance to ensure adequate calcium for bone mineralization. Severe vitamin D deficiency therefore causes rickets in children. A position paper from a working group convened by the National Osteoporosis Foundation in the US assessed the evidence for factors determining the development of peak bone mass and assigned vitamin D as moderate evidence (B grade) [11]. Achieving a high peak bone mass and preventing bone loss later in life are two strategies for reducing the risk of osteoporosis.

In tropical countries such as Malaysia, scattered epidemiologic studies have reported that 30–50% of children and adolescents have vitamin D deficiency (<30 nmol/L) and insufficiency (<50 nmol/L) [6,12,13] despite having abundant sunshine all year round. However, adequate information about vitamin D status and its determinants in growing children is limited. One study conducted among 13-year old adolescents reported a positive association between calcium and vitamin D intake with the bone mineral content of calcaneal bone [12], while a larger study among South East Asian countries showed gender, religion, and sun avoidance as risk factors for low vitamin D status in Malaysian children [13].

The aim of our study was to determine factors associated with serum 25(OH)D concentrations such as lifestyle, dietary intake, anthropometry, and body composition in 243 pre-adolescent Malaysian children from low-income families living in Kuala Lumpur. We hypothesized that the widespread prevalence of low vitamin D status in Malaysia, a country with an abundance of sunshine, is due to modifiable lifestyle factors in these children.

## 2. Methodology

### 2.1. Participants

Data were obtained from the cross-sectional analysis at baseline of 243 healthy, pre-adolescent boys and girls between 9 and 11 years old who participated in the PREBONE-KIDS study, which was a 1-year intervention study of soluble corn fiber (SCF) on bone indices in Kuala Lumpur (ClinicalTrials.gov identifier: NCT03864172). Ethical approval for the study was obtained from the Research and Ethics Committee of the International Medical University (IMU) (Trial no: R182/2016). Written informed consent was obtained from parents or legal guardians, and assent was obtained from the participants.

The study included participants who were healthy, as determined by a standard medical assessment: Tanner Stage 1 or 2 based on breast development for girls and pubic hair in boys [14], premenarcheal for girls, and able to provide assent. Participants were excluded if they had a history of serious medical conditions and received therapy with medications known to interfere with bone metabolism (e.g., steroids, hormones, diuretics, cortisone, or anti-seizure medication). Participants were recruited from 3 national primary schools around Bangsar and Brickfields, Kuala Lumpur, Malaysia (3.1390° N, 101.6869° E). A study brochure and study information sheet along with informed consent and assent forms were distributed to a total of 1293 children from Primary 3 to 5 grades through their class teachers in the schools. Screening sessions were carried out at the schools for participants whose consent had been obtained from their parents to assess eligibility.

### 2.2. General Health Screening

All recruited participants were screened for general health by the study pediatricians, and Tanner staging was obtained through self-report after verification by the study pediatricians.

### 2.3. Bone Parameters and Body Composition

A whole-body and lumbar spine (L1–L4) dual-energy X-ray absorptiometry (DXA) scan (GE Lunar iDXA, GE Healthcare LLC, Texas, USA) equipped with a pediatric software using Asian database (Lunar enCORE version 13.60.033) was performed by a trained technician to assess areal bone mineral density (BMD), bone mineral content (BMC), and body composition, i.e., fat mass (FM) (kg and percentage of body weight) and fat-free mass (FFM) (kg) from the whole body, trunk, android, and gynoid. The coefficient of variation (CV%) was 0.42% for total body BMD, 0.83% for lumbar spine BMD, 0.86% for FM, and 1.37% for FFM.

### 2.4. Serum 25-OH Vitamin D and PTH

Serum 25-OHD was determined using liquid chromatography-tandem mass spectrometry (LC–MS/MS) with an Agilent 1260 Infinity liquid chromatograph (Agilent Technologies, Waldbronn, Germany) coupled to a QTRAP^®^ 5500 tandem mass spectrometer (AB SCIEX, Foster City, CA, USA) using a MassChrom^®^ 25-OH-Vitamin D3/D2 in serum/plasma reagent kit including a 3-epi-25-OH-Vitamin D3/D2 upgrade diagnostics kit (Chromsystems, Munich, Germany). The coefficients of variation of 25-OHD_3_ and 25-OHD_2_ were 5.9% and 3.3%, respectively. The 25(OH)D_2_ and 25(OH)D_3_ levels were combined and expressed as total 25(OH)D in nmol/L. Serum iPTH was determined using direct chemiluminometric technology on the ADVIA Centaur (Siemens, Chicago, USA) with a CV of 1.67%.

### 2.5. Calcium and Vitamin D Intake

Participants were interviewed by researchers for their usual diet intakes using a 7-day diet history. Participants were asked to describe their food intake using household measurements and with the assistance of a food portion album, and these were verified with their parents or caretakers. Calcium in foods was based on values in the Nutrient Composition of Malaysian Foods [15] and nutrient labels for manufactured foods. Vitamin D content was obtained from the Singapore Food Composition Book (Ministry of Health Singapore) [16] as a primary source (due to similarity with foods consumed in Malaysia) and nutrient labels for manufactured foods. The dietary data were analyzed with the Nutritionist Pro diet analysis software (version 7.4.0, 2019, Axxya Systems LLC, WA, USA).

### 2.6. Sun Index (SI)

Participants completed a self-reported questionnaire adapted from Barger-Lux and Heaney [17] and validated by Nurbazlin et al. [18] on time and frequency spent in outdoor activities over the previous week, as well as their usual outdoor attire. The questionnaire consisted of eight activity categories (transportation to school, transportation to tuition center, club activities, home activities, school activities, activities during the recess period, and others). Information about duration (in measures of hours and days), sunscreen, umbrella usage, clothing, and hat usage for each outdoor activity performed in the past 7 days were recorded. The fraction of body surface area (BSA) exposed to sunlight with their attire was calculated using “Rule of Nine.” The Sun Index was calculated using the combined measurement of time of outdoor sunlight exposure and the fraction of BSA exposed to sunlight, i.e., Sun Index = (hours of sun exposure per week) × (fraction of BSA exposed to sunlight). Data on sun exposure were collected in the months from March to May, which were not monsoon season in Malaysia.

### 2.7. Physical Activity

Participants were interviewed for their habitual activities using a validated questionnaire (cPAQ) [19], and metabolic equivalent task (MET) scores computed by Ainsworth et al. [20] and Kemper et al. [21] were used.

### 2.8. Anthropometry

Height was measured without shoes to the nearest 0.1 cm using a stadiometer (SECA 206, Hamburg, Germany). Weight was measured to the nearest 0.1 kg with a calibrated scale (TANITA HD-301, TANITA Corporation, Japan). The referenced BMI categories were thinness (BMI Z-score: <−2.0), normal (BMI Z-score: from −2.0 to 1.0), overweight (BMI Z-score: from >1.0 to ≤2.0), and obese (BMI Z-score: >2.0) [22].

### 2.9. Statistical Analysis

Quantitative variables were described as means and standard deviations, while qualitative variables were described as frequencies and percentages. Diagnosis tests of normality, multicollinearity, and residual analysis were performed. In the analysis, *t*-test, ANOVA, chi-square test, correlation analysis, multiple linear regression analysis, and stepwise analysis were used. For all tests, the *p*-value was set as 0.05.

## 3. Results

Participants had a mean age of 10.1 ± 1.0 years (Table 1), with the majority of the Malay ethnicity and in Tanner Stage 1. The overall prevalence of overweight and obesity were 15.2% and 17.7%, respectively, with a higher prevalence of overweight in girls and a higher prevalence of obesity among boys. BMD and BMC values were significantly higher in boys compared to girls.

The overall mean serum 25(OH)D concentration was 43.9 ± 14.5 nmol/L, and girls had significantly lower mean values than boys (girls: 36.8 ± 11.9 nmol/L vs. boys: 50.3 ± 13.7 nmol/L; *p* < 0.001). Girls had significantly higher PTH levels (*p* < 0.001) than boys, spent significantly (*p* < 0.001) less hours in the sun per week compared to boys, and had less BSA exposed to sunlight (*p* < 0.001), thus contributing to their lower Sun Index value than boys (*p* < 0.001). Boys were significantly more active than girls (MET scores: 961 ± 502 for boys vs. 670 ± 317 for girls; *p* < 0.001). In terms of dietary intakes, boys had higher intakes of energy, protein, calcium, and vitamin D than girls (*p* < 0.001). The calcium and vitamin D intakes reported by both sexes were significantly less than the recommended intakes for children in this age-group (1000 mg/day for calcium and 5 µg/day for vitamin D) [23].

Table 2 shows that 18.9% of the participants had vitamin D deficiency, based on the IOM 2011 [25] cut-off value of below 30 nmol/L, with girls (35.3%) having a 9-fold higher prevalence than boys (3.9%). A total of 42.4% of the participants had a value below 40 nmol/L, with girls (62.9%) also having a higher prevalence than boys (23.6%). However, as different societies and expert bodies have defined 50 nmol/L as the “vitamin D requirement of nearly all normal healthy persons,” [1] akin to the US RDA recommendation, 69.4% of the total participants had values below 50 nmol/l (52.8% in boys and 87.9% in girls).

Bone parameters (BMD, BMC, BMD Z-scores) were found to be not significantly associated with serum 25(OH)D concentration in our population in a bivariate analysis (provided in the Supplementary Table). We then tested the associations between age, height, lean mass, fat mass, physical activity scores, calcium intake, vitamin D intake, hours of sun exposure, body surface area (BSA), Sun Index and serum 25(OH)D concentration (provided in the Supplementary Materials). Serum 25(OH)D concentration was not significantly associated with age (*p* = 0.464) and calcium intake (*p* = 0.458). Among the predictor variables, lean mass and fat mass were highly correlated (*p* = 0.837), thus indicating a problem of multicollinearity. Based on literature, only fat mass was considered in further analysis. When analyzing the interaction effects between gender and the predictor variables, the BSA*gender effect was significant (*p* = 0.001). In the initial multiple regression analysis, height, fat mass, physical activity scores, vitamin D intake, hours of sun exposure, BSA, Sun Index, and BSA*gender were tested. The results are presented in Table 3. In this analysis, only the Sun Index (*p* = 0.049) and BSA*gender (*p* = 0.015) were significantly associated with serum 25(OH)D concentrations. The R-square value was 21.4%, and the assumptions on the residuals were met.

A stepwise analysis was performed with entry and removal of 0.05 and 0.10 *p*-values. The results are shown in Table 4. In this analysis, only fat mass (*p* = 0.011), PTH (*p* < 0.001), Sun Index (*p* = 0.006), and BSA*gender (*p* < 0.001) were significantly associated with serum 25(OH)D. In terms of relative importance, BSA*gender ranked the highest (beta = 0.279), while fat mass ranked the lowest (beta = −0.149). The R-square value was 19.5%.

**Figure 1:**
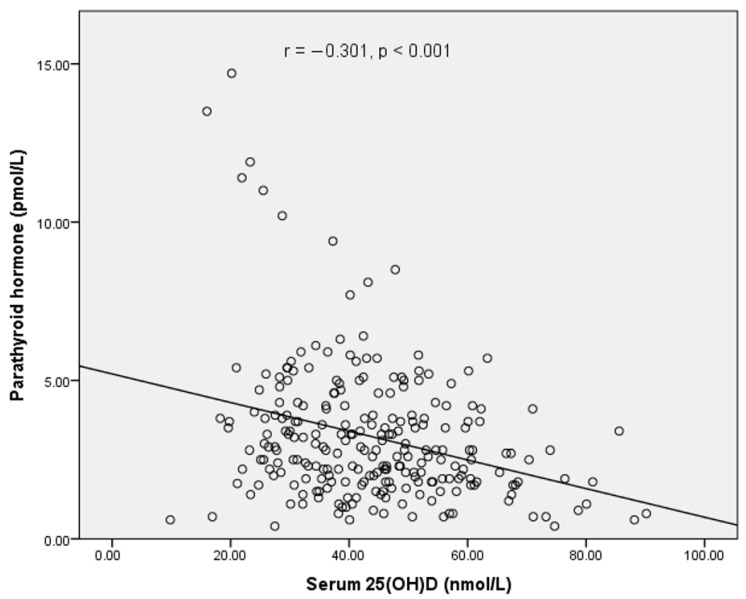
Relationship between iPTH and serum 25(OH)D concentration.

Lastly, our analysis showed an inverse significant correlation between iPTH and 25(OH)D levels (r = −0.301; *p* < 0.001), as shown in Figure 1. About 4% of the participants (8 girls and 2 boys) had an elevated iPTH concentration above the upper limit of 6.5 pmol/L, and their corresponding serum 25(OH)D concentrations were <50 nmol/L. However, we did not observe any obvious deflation or increase in iPTH in the majority of the participants, even though their 25(OH)D concentrations were below 30 nmol/L.

## 4. Discussion

This cross-sectional study indicated that among predominantly Malay pre-adolescent children, the average serum 25(OH)D value was close to the concentration recommended by the IOM/National Academy of Medicine to cover the needs of 50% of the population (EAR), with 57.6% being vitamin-D-sufficient and 18.9% being vitamin-D-deficient when using this cut-off levels. There was a high prevalence of insufficient vitamin D status, as more than two-third of the participants had levels below 50 nmol/L, which is the cut-off recommended by the Global Consensus Recommendations [5] and akin to the IOM/NAM RDA.

There is still no agreement on which serum 25(OH)D concentrations should define a sufficient vitamin D status, largely because there is a lack of evidence relating vitamin D status to functional measures. However, previous studies in Malaysia have classified vitamin D status as insufficient based on cut-off values of below 50 nmol/L. Using this cut-off for comparison, our study showed that the prevalence of poor vitamin D status was 69.5%, and this is the most recent assessment among children of this age-group in Malaysia. In 2011, Khor et al. [6] reported a similarly high prevalence of 72.4% of pre-adolescent children with serum 25(OH)D concentrations below 50 nmol/l. The Southeast Asian Nutrition Surveys (SEANUTS) conducted in Indonesia, Malaysia, Thailand, and Vietnam among 16,744 children aged 0.5–12 years reported that vitamin D insufficiency (<50 nmol/L) was noted in 40–50% of children in these countries. The results of our study further attest to the global phenomenon of the high prevalence of low vitamin D status among populations living in sub-tropical and tropical countries [26].

We hypothesized that vitamin D inadequacy would be prevalent in this population who live in a tropical climate because of modifiable lifestyle factors. We found that vitamin D status was surprisingly low for a tropical region and that the strongest predictors for vitamin D adequacy were related to estimated skin sun exposure, Sun Index (which is time spent outdoors*body surface exposed to sun), and excess body fat mass. Girls were found to have a significantly lower mean serum vitamin D levels than boys, and this coincided with significantly lower body surface area exposed to sunlight and fewer hours spent in the sun compared to boys. As our participants were mostly Malay children, the culture and religious practice of wearing head scarf, long-sleeved clothing, and long skirts or trousers contributed to the low body surface area exposed to sunlight in girls. In contrast, the boys could wear shorts or short sleeve attire, so they had greater sunlight exposure that contributed to a higher mean serum vitamin D level and a lower prevalence of vitamin D deficiency than girls. These lifestyle factors attributed to attire, culture, and religion were similarly reported by the SEANUTS study in children, as well as by studies in Malay adults [18,27].

While body surface area exposed to sunlight explains the lower vitamin D status in girls compared to the boys in our study who were less covered in terms of clothing and more active than girls, it does not completely explain why more than half of the boys still had inadequate vitamin D status. Despite having more skin area exposed to sunlight, the time spent in outdoor activities under the sun averaged seven hours a week (1 h a day) by the boys in this study, and this was lower than reported among Malaysian adolescents who spent more than 2 h on weekdays outdoors [28]. Malaysian children in a similar age as this study, i.e., aged 7–12 years, have been reported to spend an average of 6.7 h daily in indoor sedentary activities such as watching television, using the computer, and doing schoolwork [29].

Another factor associated with the low vitamin D status in the children in our study was related to their body composition. Fat mass was a significant negative predictor of serum 25(OH)D levels, although its relative importance was lower than sun exposure in our analysis. Excess body weight or fat accumulation in children and adolescents are associated with lower 25(OH)D concentrations and higher PTH concentrations [30,31,32]. The low levels of 25(OH)D in obesity have been attributed to excessive fat-soluble vitamin deposition in adipose tissue and, consequently, a decreased bioavailability of vitamin D [33]. Khor et al. [6] earlier reported that low vitamin D status among children in this age group was associated with a high BMI. Our current study findings support the indication that a greater storage capacity for vitamin D due to obesity is contributing to low vitamin D status. Nevertheless, the causality between obesity and low serum 25(OH)D concentration requires further investigation, as recent evidence has shown that obesity causes reductions in the activity of the hepatic 25-hydroxylase enzyme that converts vitamin D_3_ to 25(OH)D [34].

Both boys and girls had extremely poor intakes of below 2 µg/day for vitamin D and less than 400 mg/day for calcium. Most of the participants in this study were from urban poor families, and the assessment of their dietary intakes showed low consumption of milk, dairy foods, and fish, which are sources of vitamin D and calcium. Other studies in pre-adolescent children in urban Kuala Lumpur have also reported a mean calcium intake of less than 400 mg/day and a vitamin D intake of less than 3 µg/day [12,35]. The low vitamin D intakes in this study could have been inaccurate due to the limited food database. The local food composition tables do not contain vitamin D data, and vitamin D information were obtained from the Singapore food database, which had foods similar to Malaysia. There is an urgent need for analyzing vitamin D content in local foods given the high prevalence of vitamin D insufficiency in our population.

PTH appears to be an important variable that was inversely associated with serum 25(OH)D levels in this study. Prolonged vitamin D deficiency leads to impaired 1,25(OH)2D_3_ synthesis, and intestinal calcium malabsorption gives rise to raised iPTH levels (secondary hyperparathyroidism) [36]. However, no obvious deflation of iPTH concentrations was seen, even at very low levels of serum 25(OH)D among this population. This suggests that the lower limits of 25(OH)D levels for the definition of vitamin D deficiency and insufficiency in our population may differ from current international recommendations. Nevertheless, 4% of the children had an elevated iPTH secondary to low vitamin D status, which could later affect the degree and course of secondary hyperparathyroidism. Subclinical vitamin D insufficiency should also not be overlooked because of a normal PTH level.

We did not find any association between serum 25(OH)D and bone variables in a bivariate analysis. Previous studies have reported a positive association between BMD and serum 25(OH)D levels in early and late pubertal children [37,38,39]. However, other studies, like ours, have not found any association between vitamin D status and BMD in pre-adolescent children [40,41]. Yang et al. [42] examined the association of vitamin D status at different stages of growth with bone measures at 8, 16, and 25 years old, and they suggested that vitamin D status at the youngest age of 8 years old may not be as important as during adolescence in influencing BMD during young adulthood. The BMD and BMC of the children in this age-group could also be more easily determined by body weight and lean mass than by vitamin D status [41,43]. Hence, a prospective longitudinal study design would better verify the associations between 25(OH)D and associated changes in BMD in our population.

Our study strengths included being the first to examine predictors of vitamin D status in predominantly Malay children, who comprise an under-studied population, and reporting serum 25(OH)D levels measured using LC–MS/MS, which is considered a reference method [44]. Nevertheless, a limitation of this study was its cross-sectional nature to examine causality and the measure of sun exposure that was self-reported using questionnaire instead of using UV-sensitive badges that objectively measure actual UV-B exposure. Future studies should include other ethnic groups to elucidate whether the findings are similar in Chinese and Indian children in Malaysia.

Our study results reveal the need for strategies to combat the high prevalence of poor vitamin D status reported in this young population. Vitamin D supplementation may need to be considered to improve serum 25(OH)D levels among children who follow strict religious dress codes [40]. The fortification of foods, which has been adopted by many developed countries globally, has emerged as a safe and cost-effective strategy to control vitamin D deficiency [45] The promotion of a healthier lifestyle with increased outdoor activity and sunlight exposure while maintaining a healthy body weight among children is warranted.

## 5. Conclusions

In conclusion, the majority of predominantly Malay pre-adolescent children living in Kuala Lumpur in our study were found to have inadequate vitamin D status. The strongest predictors of serum 25(OH)D concentrations were restricted to modifiable factors of skin exposure to sunshine, time spent outdoors, and high body fat mass due to obesity. Public health guidance to encourage sunlight exposure through outdoor physical activity and achieving healthy body weight would be important to ensure optimal vitamin D status in these children.

## Figures and Tables

**Table 1:** Descriptive characteristics of participants (*N* = 243).

	Total (*n* = 243)	Boys (*n* = 127)	Girls (*n* = 116)	*p*-Value ^¥^
Age	10.1 ± 1.0	10.2 ± 0.9	10.0 ± 1.0	0.122
Ethnicity (*n*,%)				
Malay	220 (90.5)	110 (86.6)	110 (94.8)	
Indian	23 (9.5)	17 (13.4)	6 (5.2)	
Tanner stage (*n*,%)				
Stage 1	230 (94.7)	125 (98.4)	105 (90.5)	
Stage 2	13 (5.3)	2 (1.6)	11 (9.5)	
Monthly household income (*n*,%) #				0.275
Low < RM4849	183 (75.3)	100 (78.7)	83 (71.6)	
Middle income RM4850-10959	37 (15.2)	17 (13.4)	20 (17.2)	
High income ≥ RM10960	14 (5.8)	6 (4.7)	8 (6.9)	
No response	9 (3.7)	4 (3.2)	5 (4.3)	
Serum 25(OH)D (nmol/L)	43.9 ± 14.5	50.3 ± 13.7	36.8 ± 11.9	<0.001 **
PTH (pmol/L)	3.22 ± 2.14	2.81 ± 1.81	3.68 ± 2.37	0.001 *
BMI (kg/m^2^)	18.0 ± 4.5	18.3 ± 4.8	17.7 ± 4.2	0.332
BMI-for-age Z-score	0.187 ± 1.693	0.321 ± 1.742	0.040 ± 1.632	0.198
BMI Z-score classification, *n* (%)				
Thinness	21 (8.7)	9 (7.1)	12 (10.3)	
Normal	142 (58.4)	74 (58.3)	67 (57.8)	
Overweight	37 (15.2)	18 (14.2)	21 (18.1)	
Obese	43 (17.7)	26 (20.5)	16 (13.8)	
LSBMC (g)	21.7 ± 5.2	21.6 ± 4.7	21.9 ± 5.7	0.725
LSBMD (g/cm^2^)	0.725 ± 0.091	0.715 ± 0.081	0.736 ± 0.100	0.064
TBBMC (g)	1129.5 ± 231.6	1160.4 ± 237.9	1095.6 ± 220.6	0.029 *
TBBMD (g/cm^2^)	0.768 ± 0.075	0.780 ± 0.075	0.754 ± 0.072	0.006 **
TBBMD z-score	0.789 ± 0.960	0.890 ± 0.921	0.678 ± 0.994	0.087
Lean mass (kg)	21.79 ± 5.29	22.50 ± 5.40	21.00 ± 5.07	0.026 *
Fat mass (kg)	10.93 ± 7.08	11.00 ± 7.98	10.85 ± 5.98	0.876
Body fat percentage (%)	29.87 ± 8.44	28.82 ± 9.17	31.06 ± 7.39	0.035 *
Energy (Kcal/day)	1457 ± 450	1543 ± 463	1363 ± 417	0.002 *
Protein intake (g/day)	61.4 ± 21.6	65.7 ± 23.3	56.6 ± 18.7	0.001 *
Calcium intake (mg/day)	329 ± 202	355 ± 215	300 ± 185	0.035 *
Vitamin D intake (µg/day)	1.6 ± 1.6	1.8 ± 1.8	1.3 ± 1.2	0.014 *
PA level (MET scores)	822 ± 447	961 ± 502	670 ± 317	<0.001 **
Hours of sun exposure (h/week)	6.7 ± 3.4	7.4 ± 3.7	5.9 ± 2.8	<0.001 *
Body surface area exposed to sun	0.14 ± 0.08	0.20 ± 0.06	0.09 ± 0.05	<0.001 *
Sun Index	1.08 ± 0.77	1.49 ± 0.78	0.62 ± 0.44	<0.001 *

^¥^ Significance difference between groups at * *p* < 0.05; ** *p* < 0.001 by independent *t*-test or χ^2^ test for categorical data. All values are mean ± SD unless otherwise stated. # Dept of Statistics Malaysia, 2019 [24]. Abbreviations: TB: total body; LS: lumbar spine; BMD: bone mineral density; BMC: bone mineral content.

**Table 2:** Proportion of participants at various serum 25(OH)D cut-offs to define deficiency (*N* = 243).

25(OH)D Levels *	Total (*n* = 243)	Boys (*n* = 127)	Girls (*n* = 116)
<50 nmol/L	169 (69.4)	67 (52.8)	102 (87.9)
<40 nmol/L	103 (42.4)	30 (23.6)	73(62.9)
Inadequacy (30–40 nmol/L)	57 (23.5)	25 (19.7)	32 (27.6)
Deficiency (<30 nmol/L)	46 (18.9)	5 (3.9)	41 (35.3)

* Based on IOM /NAM (2011 cut-offs, *n* (%).

**Table 3:** Multiple linear regression analysis on predictors of serum 25(OH)D (*N* = 243).

Predictors	Unstandardized		Standardized	t	*p*-Value	VIF
	B	SE	Beta			
(Constant)	19.482	6.347		3.069	0.002	
Height, m	−0.029	0.050	−0.045	−0.577	0.565	1.815
Fat mass, kg	0.000	0.000	−0.109	−1.444	0.150	1.700
MET score	0.001	0.001	0.084	0.882	0.379	2.708
Vit D, µg/day	0.367	0.228	0.100	1.607	0.109	1.145
Sun exposure (h/week)	−0.234	0.182	−0.135	−1.284	0.200	3.295
BSA	−3.743	9.084	−0.049	−0.412	0.681	4.244
Sun Index	1.868	0.945	0.248	1.977	0.049	4.687
BSA*gender	15.490	6.299	0.282	2.459	0.015	3.904

**Table 4:** Stepwise regression for predictors of serum 25(OH)D (*N* = 243).

Predictors	Unstandardized		Standardized	t	*p*-Value	VIF
	B	SE	Beta			
(Constant)	15.732	0.763		20.625	0.000	
Fat mass, kg	−0.0001	0.00005	−0.149	−2.558	0.011	1.005
Sun Index	1.497	0.537	0.199	2.787	0.006	1.511
BSA*gender	15.370	3.925	0.279	3.916	0.000	1.513

## Supplementary Materials

The following are available online at https://www.mdpi.com/article/10.3390/nu13072175/s1. Table S1. Associations between 25(OH)D and bone parameters. Table S2. Associations between 25OH(D) and variables in the study (Correlation coefficients). Table S3. Associations between 25OH(D) and variables in the study (*p*-values).

## References

[1] Amrein K., Scherkl M., Hoffmann M., Neuwersch-Sommeregger S., Köstenberger M., Berisha A.T., Martucci G., Pilz S., Malle O. (2020). Vitamin D deficiency 2.0: An update on the current status worldwide. Eur. J. Clin. Nutr..

[2] Cashman K.D., Dowling K.G., Škrabáková Z., Gonzalez-Gross M., Valtueña J., De Henauw S., Moreno L., Damsgaard C.T., Michaelsen K.F., Mølgaard C. (2016). Vitamin D deficiency in Europe: Pandemic?. Am. J. Clin. Nutr..

[3] Almoudi M.M., Hussein A.S., Abu Hassan M.I., Schroth R.J. (2019). Dental caries and vitamin D status in children in Asia. Pediatr. Int..

[4] Ross A.C. (2011). The 2011 report on dietary reference intakes for calcium and vitamin D. Public Health Nutr..

[5] Munns C.F., Shaw N., Kiely M., Specker B.L., Thacher T., Ozono K., Michigami T., Tiosano D., Mughal M.Z., Mäkitie O. (2016). Global Consensus Recommendations on Prevention and Management of Nutritional Rickets. Horm. Res. Paediatr..

[6] Khor G.L., Chee W.S.S., Shariff Z.M., Poh B.K., Arumugam M., Ab Rahman J., Theobald H.E. (2011). High prevalence of vitamin D insufficiency and its association with BMI-for-age among primary school children in Kuala Lumpur, Malaysia. BMC Public Health.

[7] Poh B.K., Rojroongwasinkul N., Le Nguyen B.K., Sandjaja, Ruzita A.T., Yamborisut U., Hong T.N., Ernawati F., Deurenberg P., Parikh P. (2016). 25-hydroxy-vitamin D demography and the risk of vitamin D insufficiency in the South East Asian Nutrition Surveys (SEANUTS). Asia Pac. J. Clin. Nutr..

[8] Taylor S.N. (2020). Vitamin D in Preterm and Full-Term Infants. Ann. Nutr. Metab..

[9] Heaney R.P., Dowell M.S., Hale C.A., Bendich A. (2003). Calcium Absorption Varies within the Reference Range for Serum 25-Hydroxyvitamin D. J. Am. Coll. Nutr..

[10] Steingrimsdottir L., Gunnarsson O., Indridason O.S., Franzson L., Sigurdsson G. (2005). Relationship between serum parathyroid hormone levels, vitamin D sufficiency, and calcium intake. JAMA.

[11] Heaney R.P., Abrams S., Dawson-Hughes B., Looker A., Marcus R., Matkovic V., Weaver C. (2001). Peak Bone Mass. Osteoporos. Int..

[12] Suriawati A.A., Majid H.A., Al-Sadat N., Mohamed M.N.A., Jalaludin M.Y. (2016). Vitamin D and Calcium Intakes, Physical Activity, and Calcaneus BMC among School-Going 13-Year Old Malaysian Adolescents. Nutrients.

[13] Poh B.K., Ng B.K., Haslinda M.D.S., Shanita S.N., Wong J.E., Budin S.B., Ruzita A.T., Ng L.O., Khouw I., Norimah A.K. (2013). Nutritional status and dietary intakes of children aged 6 months to 12 years: Findings of the Nutrition Survey of Malaysian Children (SEANUTS Malaysia). Br. J. Nutr..

[14] Duke P.M., Litt I.F., Gross R.T. (1980). Adolescents’ self-assessment of sexual maturation. Pediatrics.

[15] Tee E., Ismail M., Nasir M., Idris K. (1987). Nutrient Composition of Malaysian Foods.

[16] Health Promotion Board Energy & Nutrient Composition of Food. https://focos.hpb.gov.sg/eservices/ENCF/.

[17] Barger-Lux M.J., Heaney R.P. (2002). Effects of Above Average Summer Sun Exposure on Serum 25-Hydroxyvitamin D and Calcium Absorption. J. Clin. Endocrinol. Metab..

[18] Nurbazlin M., Chee W.S.S., Rokiah P., Tan A.T.B., Chew Y.Y., Nusaibah A.R.S., Chan S.P. (2013). Effects of sun exposure on 25(OH) vitamin D concentration in urban and rural women in Malaysia. Asia Pac. J. Clin. Nutr..

[19] Nor Aini J., Poh B.K., Chee W.S.S. (2013). Validity of a children’s physical activity questionnaire (cPAQ) for the study of bone health. Pediatr. Int..

[20] Ainsworth B.E., Haskell W.L., Whitt M.C., Irwin M.L., Swartz A.M., Strath S.J., O’Brien W.L., Bassett D.R., Schmitz K.H., Emplaincourt P.O. (2000). Compendium of Physical Activities: An update of activity codes and MET intensities. Med. Sci. Sports Exerc..

[21] Kemper H., Bakker I., Twisk J., van Mechelen W. (2002). Validation of a physical activity questionnaire to measure the effect of mechanical strain on bone mass. Bone.

[22] WHO (1995). Physical Status: The Use and Interpretation of Anthropometry, Report of a WHO Expert Committee.

[23] National Coordinating Committee on Food and Nutrition, Ministry of Health Malaysia (2017). Recommended Nutrient Intakes for Malaysia. A Report of the Technical Working Group on Nutritional Guidelines.

[24] Department of Statistics Malaysia (2019). Household Income and Basic Amenities Survey (HIS) Report 2019.

[25] Rosen C.J., Abrams S., Aloia J.F., Brannon P.M., Clinton S.K., Durazo-Arvizu R.A., Gallagher J.C., Gallo R.L., Jones G., Kovacs C.S. (2012). IOM Committee Members Respond to Endocrine Society Vitamin D Guideline. J. Clin. Endocrinol. Metab..

[26] Palacios C., Gonzalez L. (2014). Is vitamin D deficiency a major global public health problem?. J. Steroid Biochem. Mol. Biol..

[27] Moy F.M. (2011). Vitamin D status and its associated factors of free living Malay adults in a tropical country, Malaysia. J. Photochem. Photobiol. B Biol..

[28] Quah S.W., Majid H.A., Al-Sadat N., Yahya A., Su T.T., Jalaludin M.Y. (2018). Risk factors of vitamin D deficiency among 15-year-old adolescents participating in the Malaysian Health and Adolescents Longitudinal Research Team Study (MyHeARTs). PLoS ONE.

[29] Lee S.T., Wong J.E., Shanita S.N., Ismail M.N., Deurenberg P., Poh B.K. (2014). Daily Physical Activity and Screen Time, but Not Other Sedentary Activities, Are Associated with Measures of Obesity during Childhood. Int. J. Environ. Res. Public Health.

[30] Barja-Fernández S., Aguilera C.M., Martínez-Silva I., Vazquez R., Gil-Campos M., Olza J., Bedoya J., Cadarso-Suárez C., Gil Á., Seoane L.M. (2017). 25-Hydroxyvitamin D levels of children are inversely related to adiposity assessed by body mass index. J. Physiol. Biochem..

[31] Asghari G., Yuzbashian E., Wagner C.L., Mahdavi M., Shamsi R., Hosseinpanah F., Mirmiran P. (2019). The relation between circulating levels of vitamin D and parathyroid hormone in children and adolescents with overweight or obesity: Quest for a threshold. PLoS ONE.

[32] Plesner J.L., Dahl M., Fonvig C.E., Nielsen T.R.H., Kloppenborg J.T., Pedersen O., Hansen T., Holm J.-C. (2018). Obesity is associated with vitamin D deficiency in Danish children and adolescents. J. Pediatr. Endocrinol. Metab..

[33] Wortsman J., Matsuoka L.Y., Chen T.C., Lu Z., Holick M.F. (2000). Decreased bioavailability of vitamin D in obesity. Am. J. Clin. Nutr..

[34] Bouillon R., Bikle D. (2019). Vitamin D Metabolism Revised: Fall of Dogmas. J. Bone Miner. Res..

[35] Yang W., Burrows T., MacDonald-Wicks L., Williams L., Collins C., Chee W. (2016). The Family Diet Study: A cross-sectional study into the associations between diet, food habits and body weight status in M alay families. J. Hum. Nutr. Diet..

[36] Uday S., Hoegler W. (2017). Nutritional Rickets and Osteomalacia in the Twenty-first Century. Curr. Osteoporos. Rep..

[37] Sharawat I.K., Dawman L. (2019). Bone mineral density and its correlation with vitamin D status in healthy school-going children of Western India. Arch. Osteoporos..

[38] Esterle L., Nguyen M., Walrantâ Debray O., Sabatier J., Garabedian M. (2010). Adverse interaction of low calcium diet and low 25 (OH) D levels on lumbar spine mineralization in latepubertal girls. J. Bone Miner. Res..

[39] Pekkinen M., Viljakainen H., Saarnio E., Lamberg-Allardt C., Mäkitie O. (2012). Vitamin D Is a Major Determinant of Bone Mineral Density at School Age. PLoS ONE.

[40] Hatun S., Islam O., Cizmecioglu F., Kara B., Babaoglu K., Berk F., Gökalp A.S. (2005). Subclinical Vitamin D Deficiency Is Increased in Adolescent Girls Who Wear Concealing Clothing. J. Nutr..

[41] White Z., White S., Dalvie T., Kruger M.C., Van Zyl A., Becker P. (2019). Bone Health, Body Composition, and Vitamin D Status of Black Preadolescent Children in South Africa. Nutrients.

[42] Yang Y., Wu F., Winzenberg T., Jones G. (2019). The Association of Vitamin D in Youth and Early Adulthood with Bone Mineral Density and Microarchitecture in Early Adulthood. Calcif. Tissue Int..

[43] Baptista F., Barrigas C., Vieira F., Santa-Clara H., Homens P.M., Fragoso I., Teixeira P.J., Sardinha L.B., Vieira M.F. (2011). The role of lean body mass and physical activity in bone health in children. J. Bone Miner. Metab..

[44] Black L.J., Anderson D., Clarke M.W., Ponsonby A.-L., Lucas R.M. (2015). Analytical Bias in the Measurement of Serum 25-Hydroxyvitamin D Concentrations Impairs Assessment of Vitamin D Status in Clinical and Research Settings. PLoS ONE.

[45] Pilz S., März W., Cashman K.D., Kiely M.E., Whiting S.J., Holick M.F., Grant W.B., Pludowski P., Hiligsmann M., Trummer C. (2018). Rationale and Plan for Vitamin D Food Fortification: A Review and Guidance Paper. Front. Endocrinol..

