# Supplementary material for: Vitamin D Status Is Associated with Modifiable Lifestyle Factors in Pre-Adolescent Children Living in Urban Kuala Lumpur, Malaysia
Source: Nutrients. 2021 Jun 24;13(7):2175. doi: 10.3390/nu13072175 (PMC8308301; doi:10.3390/nu13072175)
Supplement: Supplementary file 1 [file nutrients-13-02175-s001.zip › nutrients-1221624-supplementary.pdf]

nutrients

Supplementary Materials

Vitamin D Status Is Associated with Modifiable Lifestyle Factors in Pre-Adolescent Children Living in Urban Kuala Lumpur, Malaysia

Winnie Siew Swee Chee [1,*], Chung Yuan Chang [1], Kanimolli Arasu [1], Soon Yee Wong [1], Shu Hwa Ong [1], Wai Yew Yang [1], Megan Hueh Zan Chong [1], Meenal Mavinkurve [2], Erwin Jiayuan Khoo [2], Karuthan Chinna [3] and Connie M. Weaver [4]

Table S1. Associations between 25(OH)D and bone parameters.

	Variables	25OH(D)	1	2	3	4	5	6	7
1.	TBBMD	0.062	1.000	.					
2.	TBLHBMD	−0.002	0.943**	1.000					
3.	TBBMC	0.059	0.879**	0.937**	1.000				
4.	TBLHBMC	−0.068	0.843**	0.944**	0.979**	1.000			
5.	LSBMD	−0.084	0.774**	0.791**	0.750**	0.732**	1.000		
6.	LSBMC	−0.012	0.777**	0.853**	0.889**	0.888**	0.867**	1.000	
7.	BMD Z-score	−0.041	0.902**	0.819**	0.724**	0.681**	0.710**	0.648**	1.000

Correlation coefficients, ***p* = 0.000.

Table S2. Associations between 25OH(D) and variables in the study (Correlation coefficients).

	Variables	25OH(D)	1	2	3	4	5	6	7	8	9	10
1.	Age	0.006	1.000									
2.	Height	−0.090	0.611	1.000								
3.	Lean Mass	−0.045	0.472	0.860	1.000							
4.	Fat Mass	−0.122	0.242	0.643	0.837	1.000						
5.	PAQ.MET	0.262	0.178	0.185	0.213	0.091	−0.161					
6.	Calcium	0.007	−0.066	−0.080	−0.048	−0.009	−0.033	1.000				
7.	Vit D	0.211	−0.052	−0.177	−0.154	−0.115	−0.205	0.266	1.000			
8.	Hours of sun exp	0.173	0.076	0.127	0.138	0.044	−0.084	0.054	0.190	1.000		
9.	BSA	0.338	0.138	0.106	0.125	0.072	−0.165	0.041	0.166	0.171	1.000	
10.	Sun Index	0.351	0.110	0.149	0.167	0.057	−0.151	0.042	0.213	0.743	0.573	1.000

Among the tested variables, *p*-values for all variables, except for Age and Calcium were < 0.25.
The association between Lean Mass and Fat Mass was high (r = 0.837), indication a possible multicollinearity. In the analysis only Fat Mass was included.

Table S3. Associations between 25OH(D) and variables in the study (*p*-values).

	Variables	25OH(D)	1	2	3	4	5	6	7	8	9	10
1.	Age	0.464	.									
2.	Height	0.081	0.000	.								
3.	Lean Mass	0.243	0.000	0.000	.							
4.	Fat Mass	0.029	0.000	0.000	0.000	.						
5.	PAQ.MET	0.000	0.003	0.002	0.000	0.079	0.006					
6.	Calcium	0.458	0.153	0.108	0.228	0.443	0.303	.				
7.	Vit D	0.000	0.209	0.003	0.008	0.037	0.001	0.000	.			
8.	Hours of sun exp	0.004	0.121	0.024	0.016	0.250	0.095	0.201	0.001	.		
9.	BSA	0.000	0.016	0.050	0.026	0.132	0.005	0.262	0.005	0.004	.	
10.	Sun Index	0.000	0.044	0.010	0.005	0.189	0.009	0.258	0.000	0.000	0.000	.

Among the tested variables, *p*-values for all variables, except for Age and Calcium were < 0.25.
The association between Lean Mass and Fat Mass was high (r = 0.837), indication a possible multicollinearity. In the analysis only Fat Mass was included.
